# Modulation of attractive salt taste in *Drosophila*

**DOI:** 10.1016/j.isci.2026.115128

**Published:** 2026-02-24

**Authors:** Sasha A.T. McDowell, Jinfang Li, Michael D. Gordon

**Affiliations:** 1 Department of Zoology, Life Sciences Institute, and Djavad Mowafaghian Centre for Brain Health, University of British Columbia, Vancouver, BC, Canada

**Keywords:** neurogenetics, neuroscience, sensory neuroscience

## Abstract

Modulating the palatability of salt is one way that animals regulate salt intake to promote fluid and ionic balance. In *Drosophila melanogaster*, low sodium attraction is primarily driven by “sweet” taste neurons that express the sodium-specific receptor IR56b. Here, we show that this appetitive sodium taste pathway is essential for tuning sodium attraction in response to prior salt consumption. Using *in vivo* calcium imaging, we find that a salt-enriched diet strongly suppresses the activity of IR56b neurons to salt but not to sucrose stimulation, demonstrating the existence of a sodium-specific modulatory mechanism in these cells. This effect is mediated by interoceptive mechanisms rather than sensory adaptation and does not depend on IR56b transcriptional regulation or differences in translational readthrough of a premature termination codon in the *IR56b* gene. This research provides a cellular basis for appetitive salt taste modulation and insight into mechanisms of salt homeostasis in the fly.

## Highlights

• A salt-enriched diet dramatically dampens appetitive salt taste in flies
• Direct injection of a salt-containing buffer replicates this effect
• Modulation appears specific to the salt receptor IR56b
• Modulation does not depend on transcriptional regulation of IR56b

## Graphical abstract

**Figure:**
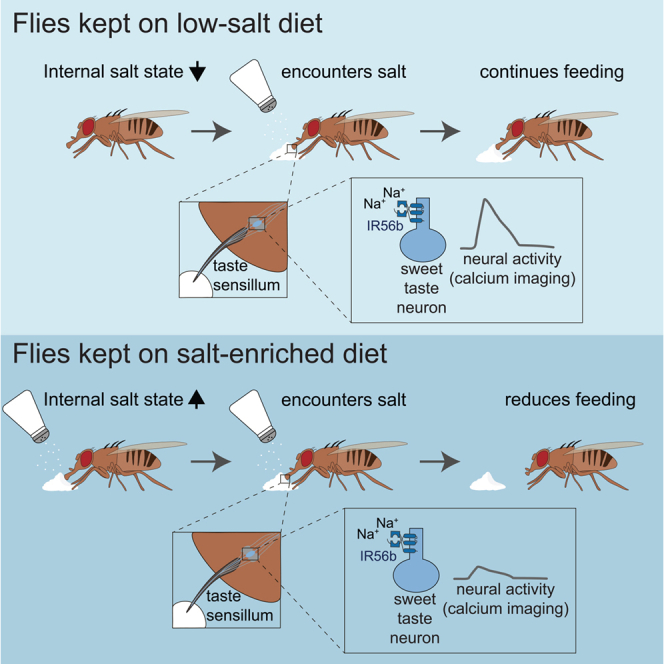

## Introduction

Animals need to regulate internal sodium levels for various physiological processes, including neuronal and muscle excitability, nutrient absorption and reabsorption, and maintaining total body fluid volume.^1^ When salt levels are dysregulated, it can negatively impact health and fitness. For example, salt overconsumption increases blood pressure and heightens the risk of cardiovascular disease in humans.^2^ Therefore, it is critical to understand the mechanisms of salt homeostasis.

One way the body can maintain salt balance is by modulating salt palatability.^3^ In mammals, there is central regulation of sodium appetite. The hormones aldosterone and angiotensin II signal low sodium in the body.^4^^,^^5^ Neurons in the nucleus of the solitary tract^6^^,^^7^^,^^8^^,^^9^ and circumventricular organs^10^^,^^11^^,^^12^ have been implicated in sensing these hormones and controlling sodium intake. However, it is unclear how these circuits interact with salt-specific taste pathways.

Several principles of salt taste detection have been elucidated in *Drosophila melanogaster*. The primary taste organ, the labellum, has hair-like structures termed sensilla that are innervated by gustatory receptor neurons (GRNs). Sweet GRNs, labeled by gustatory receptor 64f (Gr64f) sense sodium and promote its consumption.^13^ The sodium sensor within these GRNs was discovered to be a complex of ionotropic receptors (IRs): IR56b, IR25a, and IR76b.^13^^,^^14^^,^^15^^,^^16^ Aversion to high concentrations of sodium and potassium salt is mediated by bitter GRNs and a distinct population of GRNs expressing the cation non-selective salt sensor IR7c.^13^^,^^17^ Together, these three pathways mediate attraction to concentrations of sodium less than about 100 mM, and avoidance of salt concentrations above about 250 mM.^1^^,^^15^

There is abundant evidence that fly taste pathways are modulated based on various internal states.^18^^,^^19^ For example, caloric deprivation enhances the sugar sensitivity of sweet GRNs and reduces bitter GRN sensitivity while protein deprivation increases appetitive taste responses to yeast.^20^^,^^21^^,^^22^^,^^23^^,^^24^ Salt sensitivity increases in female flies after mating, and salt-deprivation triggers salt intake in flies lacking appetitive salt taste.^25^^,^^26^ However, little is known about how salt taste is modulated by prior salt intake. Our past work suggests that neurons downstream of IR7c GRNs are modulated based on salt need.^13^ Nevertheless, *IR7c* mutant flies show diet-induced modulation of their sodium consumption but not their potassium intake.^17^ This implies that modulation of appetitive sodium taste occurs, implicating the involvement of sweet GRNs.

In this study, we show that flies kept on salty food show reduced salt attraction and attenuated Gr64f GRN salt responses. Abdominal salt injection replicates this effect, while non-sodium activation of sweet GRNs does not, demonstrating an interoceptive mechanism rather than neuronal adaptation. Our evidence points toward regulation of IR56b function independently of *IR56b* transcription or translational readthrough of a premature stop codon. These findings describe a cellular mechanism for salt homeostasis and provide a foundation for future studies on how sodium taste is modulated.

## Results

### The attractive sodium pathway is modulated by salt diet

**Figure 1:**
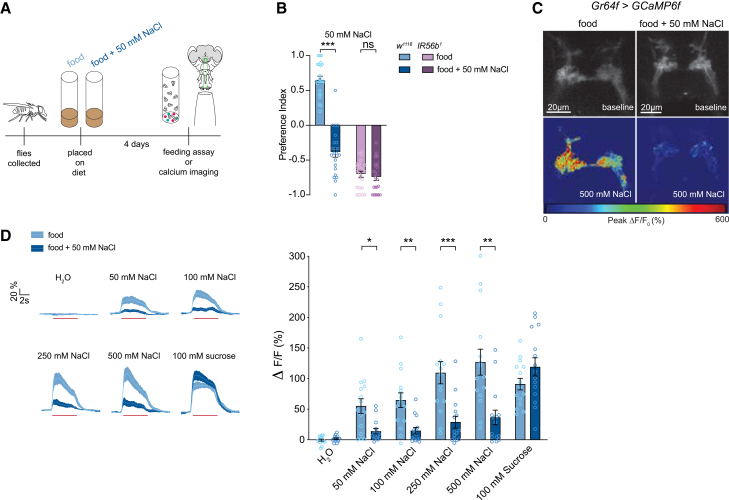
Salt diet decreases Gr64f salt responses (A) Schematic of salt conditioning prior to binary choice assay or calcium imaging. (B) Preference indices of control flies (blue) and *IR56b* mutant flies (purple). Positive values indicate preference for 50 mM NaCl + 5 mM sucrose, negative values indicate preference for 5 mM sucrose. *n* = 20 groups of ∼10 flies each. (C) Heatmaps showing *Gr64f>GCaMP6f* GRNs stimulated with 500 mM NaCl in control (left) and salt fed (right) flies. Scale bars indicate 20 μm. (D) Traces (left) and peak values (right) of *Gr64f>GCaMP6f* GRN responses to increasing NaCl concentration in control (light blue) and salt fed (dark blue) flies. *n* = 15 per group. Trace lines and shaded regions represent mean ± SEM. Red lines underneath traces indicate the timing of a 5-s stimulation. Bars represent mean ± SEM with circles indicating the individual replicates. Asterisks indicate significant difference between groups by two-way ANOVA with Sidak’s post hoc test (B) or with multiple *p* value adjusted Mann-Whitney tests performed to identify where differences between groups lay (D). ∗*p* < 0.05, ∗∗*p* < 0.01, ∗∗∗*p* < 0.001.

To measure how salt consumption impacts feeding preference for low concentrations of sodium we placed flies on the standard cornmeal/control diet we termed “food,” or the same diet supplemented with salt, termed “food + 50 mM NaCl” for four days prior to behavioral testing. Standard cornmeal-based fly food has no sodium added but may contain approximately 3 mM of sodium derived from sodium-containing ingredients.^27^ The addition of 50 mM salt was chosen since prior studies have shown this concentration to be attractive to flies^14^^,^^15^ and therefore would not inhibit ingestion of the salt-enriched food. We then conducted binary-choice assays to assess flies’ preference for 50 mM NaCl (Figure 1A). As expected, wild-type flies kept on standard food showed a preference for salt, but salt-fed flies displayed mild aversion (Figure 1B). *IR56b*^*1*^ mutants, which have lost virtually all appetitive salt taste (Figure S1),^14^ show aversion to salt. However, both control and salt-fed diets produced similar levels of aversion in *IR56b* mutants, suggesting that the attractive salt taste pathway is necessary for modulating attraction to sodium in this assay (Figure 1B).

Modulation of the attractive salt pathway could occur at the level of either the primary neuron or higher-order neurons in the circuit. Since optogenetic activation of Gr64f GRNs causes similar levels of attraction under salt fed and salt deprived conditions,^13^ we hypothesized that modulation occurs at the sensory neuron level and is overridden by optogenetic activation of those GRNs. To test this, we placed flies on the control or salt-enriched diet, expressed GCaMP6f under the control *Gr64f*^*LexA*^, and imaged the calcium responses of GR64f GRN axon terminals in the subesophageal zone (SEZ) (Figure 1C). Stimulation of the labellum for 5 s with a range of NaCl concentrations revealed that flies placed on the salt-enriched diet exhibit significantly lower Gr64f GRN salt responses (Figure 1D). This was true in mated females, males (Figure S2A), and virgin females (Figure S2B). Interestingly, this modulation was specific to salt taste, since sucrose activity remained largely unchanged between diets. Therefore, a salt-specific mechanism exists to modulate GR64f GRN activity in response to diet.

### An interoceptive sodium-specific signal modulates Gr64f salt responses

**Figure 2:**
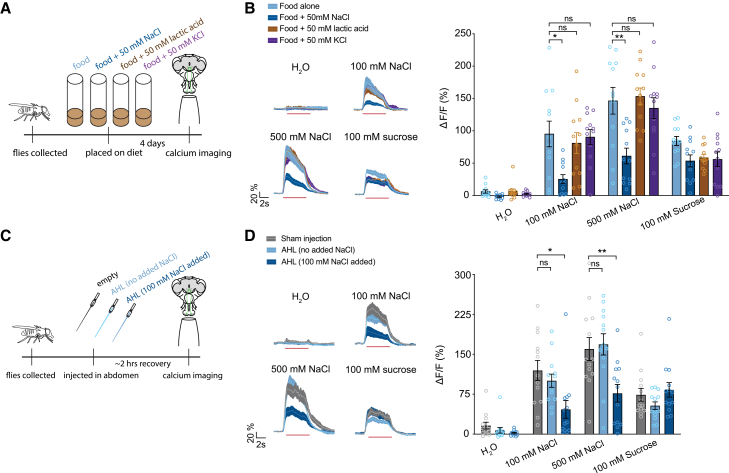
Sodium sensed internally reduces Gr64f salt responses (A) Schematic of diet conditioning prior to calcium imaging. (B) Traces (left) and peak values (right) of *Gr64f>GCaMP6f* GRN responses to NaCl in control (light blue), sodium fed (dark blue), lactic acid fed (brown), and potassium fed (purple) flies. *n* = 12 per group. (C) Schematic of various injections to the abdomen of the fly prior to calcium imaging. (D) Traces (left) and peak values (right) of *Gr64f>GCaMP6f* GRN responses to NaCl in flies with sham injections (gray), AHL with no NaCl injections (light blue) and AHL with 100 mM NaCl injections (dark blue). *n* = 13 per group. Calcium imaging traces and shaded regions represent mean ± SEM. Red lines underneath traces indicate the timing of a 5-s stimulation. Bars represent mean ± SEM with circles indicating the individual replicates. Asterisks indicate significant difference between groups by two-way repeated measures ANOVA with Sidak’s post hoc test (C); p∗<0.05 p∗∗<0.01, p∗∗∗<0.001.

Olfactory receptor neurons are known to adapt to background odors,^28^ raising the possibility that adaptation of Gr64f GRNs occurs on the salt-enriched diet due to chronically higher levels of stimulation compared to controls on the standard diet. To test this, we placed flies on food supplemented with 50 mM lactic acid, which also stimulates Gr64f salt-sensing neurons via an IR complex^29^^,^^30^ (Figures 2A and S3A). Supplemental lactic acid did not affect the subsequent sodium responses of Gr64f neurons, suggesting that reduced sodium responses are not due to general adaptation (Figure 2B).

We also wondered whether another salt, KCl, which does not activate Gr64f neurons but can affect ionic homeostasis, might elicit modulation of the sodium responses of these neurons (Figure 2A). However, a KCl-supplemented diet did not influence Gr64f neuronal sodium responses, indicating that reduced sodium responses are specific to prior sodium consumption (Figure 2B).

To confirm that salt taste modulation reflects a change in internal state, we injected the abdomens of flies with adult hemolymph-like (AHL) solution containing 100 mM NaCl or a control solution that was identical with the exception of being NaCl-free (Figure 2C). Flies injected with AHL containing 100 mM NaCl showed significantly lower sodium responses compared to sham-injected or NaCl-free AHL controls (Figure 2D). Taken together, our results indicate that Gr64f GRN salt responses are modulated by interoceptive detection of sodium state.

### A salt-enriched diet specifically affects sodium taste

**Figure 3:**
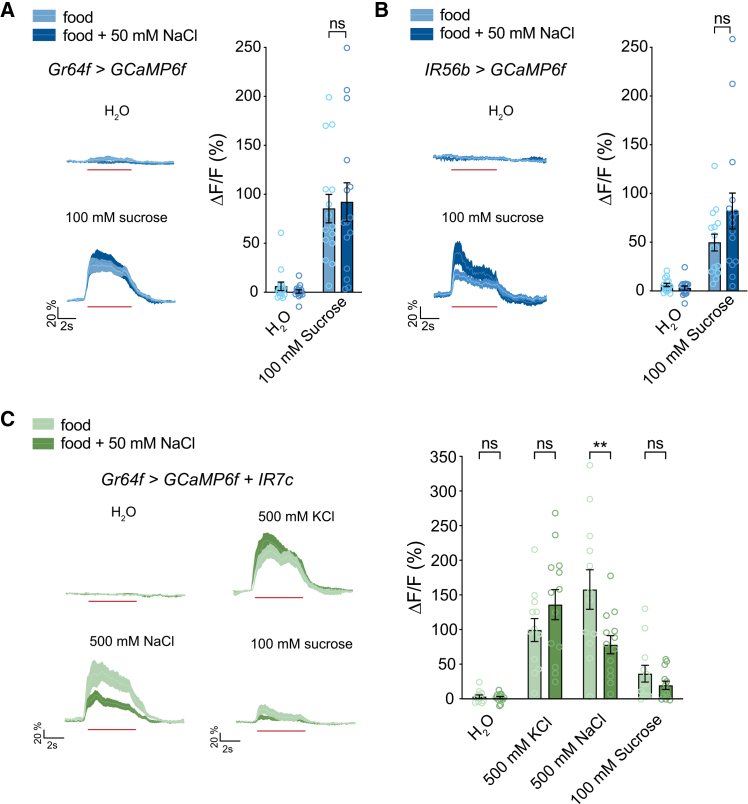
Modulation occurs by a sodium-specific molecular mechanism (A) Calcium imaging of *Gr64f>GCaMP6f* GRN responses to water and sucrose showing time course (left) and peak fluorescence changes (right). *n* = 15 flies per group. (B) Calcium imaging of *IR56b>GCaMP6f* GRN responses to water and sucrose showing time course (left) and peak fluorescence changes (right). *n* = 15 flies per group. (C) Gr64f GRN salt responses in flies expressing *IR7c* under the control of *Gr64f-Gal4* showing time course (left) and peak fluorescence changes (right). *n* = 12–13 flies per group. Calcium imaging traces and shaded regions represent mean ± SEM. Red lines underneath traces indicate the timing of a 5-s stimulation. Bars represent mean ± SEM with circles indicating the individual replicates. Asterisks indicate significant difference between groups by Welch’s two-tailed *t* test (A), Mann-Whitney test (B), or two-way repeated measures ANOVA with Sidak’s post hoc test (C). ∗∗*p* < 0.01, “ns” = not significant.

Since appetitive salt taste is mediated by sensory neurons that also participate in sugar taste, we next considered the question of how salt diet specifically affects salt taste and not sugar taste. We began by independently confirming that salt diet does not affect sucrose-evoked activity across the Gr64f GRN population (Figure 3A). Although this suggests specificity for salt, it was formally possible that modulation occurs only in the salt-sensitive subpopulation of Gr64f neurons expressing IR56b, and any differences in sugar responses in those neurons are masked by lack of modulation in the non-IR56b Gr64f GRNs. However, this possibility was ruled out by imaging IR56b GRNs, which exhibit sucrose responses that are not reduced by the salt-enriched diet (Figure 3B).

In order to gain further evidence that salt modulation is not simply a change in overall neuron excitability or synaptic efficiency, we misexpressed IR7c in Gr64f GRNs, which confers potassium responses in addition to the sodium responses found under normal conditions.^17^ We found the conferred KCl responses to Gr64f GRNs that were not modulated by salt diet, even though the endogenous sodium responses were (Figure 3C). Similar results were seen from expressing IR7c with IR56b-Gal4, which also showed no modulation of the potassium response (Figure S4). Therefore, the mechanism of sweet neuron modulation in response to a salt diet is a sodium-specific molecular mechanism. Among other implications, this result indicates that sodium modulation is not achieved via changes in the IR25a or IR76b coreceptors, which are shared between the IR56b and IR7c receptor complexes.^14^^,^^17^

### Bypassing IR56b’s transcriptional regulation and premature termination codon does not affect modulation

**Figure 4:**
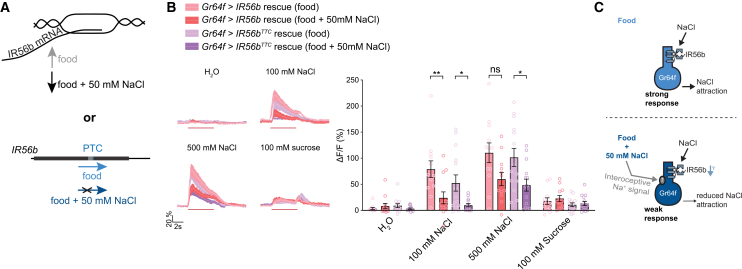
IR56b’s transcriptional regulation and PTC alone do not affect modulation (A) Possible mechanisms for IR56b regulation by salt diet: transcription (top) or translational readthrough of a premature termination codon (bottom). (B) Calcium imaging of Gr64f GRNs in *IR56b* mutants that have been rescued by *IR56b* (pink) or *IR56b*^*TTC*^ (replacement of premature termination codon with a TTC codon; purple) under the control of *Gr64f-Gal4*. *n* = 12–14. Calcium imaging traces and shaded regions represent mean ± SEM. Red lines underneath traces indicate the timing of a 5-s stimulation. Bars represent mean ± SEM with circles indicating the individual replicates. Asterisks indicate significant difference between groups by two-way ANOVA with Sidak’s post hoc test; p∗<0.05, p∗∗<0.01. (C) Schematic illustrating the modulation of attractive salt taste.

Our results indicate that modulation of salt attraction occurs at the level of sodium detection by sweet GRNs, making changes in IR56b itself the most likely mechanism. We therefore tested two possible mechanisms for IR56b modulation: transcriptional downregulation in response to a salt-enriched diet; and salt-induced changes in translational readthrough of *IR56b*’s previously described premature termination codon (PTC)^14^ (Figure 4A). First, we rescued *IR56b*^*1*^ mutants by driving *IR56b* under the control of *Gr64f-Gal4*, rather than *IR56b-Gal4*. Gr64f transcription is unlikely to be regulated by salt, given that a salt-enriched diet evokes neither a decrement in sucrose responsiveness (Figure 3A), nor a change in optogenetic response when *CsChrimson* is driven by *Gr64f-Gal4*.^13^ Thus, we would expect any transcriptional regulation of IR56b by salt diet to be bypassed. Additionally, we rescued with either wild-type *IR56b* or *IR56b*^*TTC*^, where *IR56b*’s PTC is replaced with phenylalanine encoding codon. In the latter case, we are effectively bypassing both transcriptional regulation and any potential translational control via the PTC. Nevertheless, we still observed modulation of NaCl responses following a salt-enriched diet (Figure 4B). Therefore, the detection of sodium by IR56b is likely regulated by a yet unidentified mechanism.

## Discussion

### Multiple modulatory mechanisms of sodium taste modulation

We found that adding salt to a fly’s diet for 4 days produces an interoceptive signal that suppresses its appetitive sensory response to sodium (Figure 4C). The interoceptive nature of this signal stands in contrast to a recent study showing that silencing motor neurons necessary for ingestion had no effect on the reduced sodium preference seen in flies exposed to a salt-enriched diet. This suggests that direct exposure of sweet GRNs to salt, rather than salt ingestion, also drives peripheral desensitization.^27^ Thus, it appears that both peripheral salt exposure and elevated internal salt levels are sufficient to trigger sensory modulation, suggesting that multiple modulatory mechanisms exist. Jin and colleagues^27^ found that knockdown of genes involved in clathrin-mediated endocytosis, such as *Shibire* and *clathrin*, abolished the salt desensitization they characterized, as did knockdown of *CtBP*, a key regulator of micropinocytosis.^27^ Therefore, peripheral salt exposure may trigger endocytosis, while rising internal sodium also affects sodium taste modulation via either a related or independent mechanism. There is precedent for different modulatory mechanisms producing the same effect. For sweet taste modulation, dopamine/ecdysteroid receptor (DopEcR) knockdown eliminates starvation-enhanced PER only at 6 h of starvation but not at 24 h.^22^ Dopamine is therefore required for increasing sugar sensitivity at the early stages of starvation, but other mechanisms are at play in the later stage.^22^ Thus, although we found blocking transcriptional regulation or translational control through the PTC had no effect on the modulation Gr64f neuronal sodium responses (Figure 4B), the partial redundancy with sodium-induced endocytosis could have masked their effect in our assay.

The effects of peripheral salt exposure reported by Jin and colleagues appear to be less specific to salt taste, as *CtBP* knockdown abolished salt diet-induced sugar desensitization in female flies.^27^ This is in contrast to the effect of salt in our assay, which showed sugar responses were unchanged in flies fed a salt-enriched diet (Figure 3A). Perhaps high osmolarity and/or salt stress in the sensillar lymph triggers non-specific receptor internalization. As internal sodium rises with further consumption, an interoceptive signal likely drives changes in the detection of sodium by the IR56b receptor, as our evidence points to IR56b-specific modifications (Figure 3C). While the mechanistic details behind these effects remain unclear, one possibility is involvement of cellular stress-induced pathways, like the p38 mitogen-activated protein kinase pathway.^31^^,^^32^ It would also be interesting to know whether IR56b, like the iGluRs from which IRs derive, are sensitive to cellular calcium dynamics and the activity of downstream kinases or phosphatases, which in turn may regulate receptor trafficking akin to what is seen with synaptic plasticity.^33^^,^^34^

In addition to multiple mechanisms operating to tune salt taste in the labellum, salt consumption also relies on salt detection across other taste organs across the flies’ body. These include the legs and pharyngeal sense organs, all of which contain salt sensitive neurons.^35^^,^^36^ It would be interesting to know whether the same salt modulation mechanisms operate in these other tissues.

### The site of diet-induced modulation in salt taste circuits

Regardless of the specific triggering mechanism, one interesting aspect of sodium-induced peripheral desensitization of salt taste is its restriction to appetitive GRNs. Indeed, Ppk23/IR7c-expressing high salt GRNs housed in many of the same sensilla as Gr64f GRNs are not directly modulated by salt diet.^13^ Instead, aversive responses to high salt taste appear to be modulated in downstream circuits.^13^^,^^17^ Nonetheless, modulation at the sensory level is not uncommon in fly taste, as sucrose responses of sweet neurons are modulated by energy state via DopEcR,^21^^,^^22^ and bitter neuron responses are modulated by octopamine and tyramine via the octopamine/tyramine receptor, Oct-TyrR.^23^ However, there are many accounts of modulation occurring further downstream in taste circuits, including several reports of sugar-responsive central neurons that are modulated by energy state.^37^^,^^38^^,^^39^^,^^40^^,^^41^

There are theoretical tradeoffs between regulation at the sensory neuron level versus farther downstream. On the one hand, regulation in sensory neurons has potential for a broader impact on behavior, since all downstream circuits will be affected. In this light, perhaps it is beneficial to modulate all behaviors associated with sodium attraction, while the modulation of sodium avoidance requires a more fine-tuned approach. On the other hand, the sharing of sensory neurons between modalities can necessitate sensory neuron modulation in order to achieve nutrient-specific drives. Since sodium attraction is mediated by neurons that also drive feeding in response to sugars, modulation occurring at the whole neuron level or in downstream circuits would be predicted to affect sensitivity to both salt and sugars, leading to imprecise control over nutrient intake. This is not an issue with IR7c-mediated taste because, to our knowledge, these neurons are dedicated to high salt detection.

Altogether, this study adds to the growing body of research on salt taste modulation. We find that internal sodium can reduce appetitive sodium responses via a sodium-specific molecular mechanism. Further studies are needed to assess whether IR56b is specifically internalized under salt-enriched diets and to pinpoint the source of the interoceptive signal relayed to appetitive salt taste neurons.

### Limitations of the study

Our study focuses on examining the contribution of salt-sensitive Gr64f GRNs to salt taste modulation. However, this does not preclude the involvement of other GRNs in modulating salt taste attraction, such as water-sensing GRNs that are known to have enhanced water responses when hemolymph osmolality rises.^37^ Furthermore, salt-feeding behavior relies on various organs including the legs and pharynx, where GRNs mays also exhibit modulated responses; however, this examination was beyond the scope of our study. Additionally, we used calcium signals at GRN axon terminals to assess neuronal activity, which can differ from electrophysiological recordings at the cell dendrite or soma for both technical and biological reasons. Caution should therefore be exercised when comparing our result to those using different methods.^27^ Finally, our buffer injections showed that changes in internal salt state are sufficient to modulate appetitive salt taste, but we did not demonstrate necessity. Thus, distinct mechanisms evoked by sensory experience likely also work in parallel.^27^

## Resource availability

### Lead contact

Any requests for more information, resources and reagents should be directed to and fulfilled by the lead contact, Michael D. Gordon (michael.gordon@ubc.ca).

### Materials availability

Any reagents generated from this study are available from the lead contact with no restriction.

### Data and code availability

• Raw data is publicly available on Mendeley Data: https://doi.org/10.17632/snmwf3nszs.1.
• This paper does not report original code.
• Any additional information required to reanalyze the data reported in this paper is available from the lead contact upon request.

## Acknowledgments

We thank Sanam Farman for pilot binary choice feeding assays and Gordon lab members for comments on the manuscript. Additionally, we are grateful to Dr. Eric Jan for use of the PV830 Pneumatic PicoPump. Lastly, we thank the Bloomington Stock Center and Dr. John Carlson for crucial fly stocks. This work was funded by the Natural Sciences and Engineering Research Council (10.13039/501100000038NSERC) (RGPIN-2016-03857).

## Author contributions

S.A.T.M. and M.D.G. conceived the project and wrote the manuscript. S.A.T.M. performed all experiments except for data presented in Figures 1B and S4, which were performed by J.L. and M.D.G. supervised the project.

## Declaration of interests

The authors declare no competing interests.

## STAR★Methods

### Key resources table

**Table:** 

REAGENT or RESOURCE	SOURCE	IDENTIFIER
**Chemicals, peptides, and recombinant proteins**		

Sucrose	Sigma-Aldrich	Cat# S7903
NaCl	Sigma-Aldrich	Cat# S7653
KCl	Sigma-Aldrich	Cat# P9541
CaCl_2_	BDH chemicals	Cat# BDH4524
MgCl_2_	BDH chemicals	Cat# BDH0244
Lactic Acid	Sigma-Aldrich	Cat# 69785
Agar	Sigma-Aldrich	Cat# A1296
Erioglaucine, FD&C Blue #1	Spectrum	Cat# FD110
Amaranth FD&C Red #2	Sigma-Aldrich	Cat# A1016
HEPES	Fisher Scientific	Cat# BP310100
Sodium Phosphate	BDH Chemicals	Cat# BDH9298
Sodium Bicarbonate	BDH Chemicals	Cat# BDH9280
D-ribose	Sigma-Aldrich	Cat# R7500
Active dry yeast	Fleischmann	Cat# 2192
Corn Meal	Anita’s Mill	Cat# 18006
MgSO_4_.7H_2_O	BioShop	Cat# 10034-99-8
CaCl_2_.2H_2_O	BioShop	Cat# 10035-04-8
Dextrose	Tate & Lyle	Cat# DE22K92752
Sugar	Rogers Lantic	Cat# 25200
Phosphoric acid	Anachemia	Cat# 7664-38-2
Propionic acid	J.T. Baker	Cat# 79-09-4

**Deposited data**		

Raw data from all Figures	Mendeley Data	https://doi.org/10.17632/snmwf3nszs.1

**Experimental models: Organisms/strains**		

*D. melanogaster: IR56b*^*1*^	Dweck et al.^14^	NA
*D. melanogaster: IR56b-Gal4*	Dweck et al.^14^	NA
*D. melanogaster: UAS-IR56b*	Dweck et al.^14^	NA
*D. melanogaster: UAS-IR56b*^*TTC*^	Dweck et al.^14^	NA
*D. melanogaster: w*^*1118*^	McDowell et al.^17^	NA
*D. melanogaster: UAS-IR7c*	McDowell et al.^17^	NA
*D. melanogaster: LexAOp-GCaMP6f*	Bloomington Drosophila Stock Center	BDSC: 44277; RRID: BDSC_44277
*D. melanogaster: LexAOp-GCaMP6m*	Bloomington Drosophila Stock Center	BDSC: 44275; RRID: BDSC_ 44275
*D. melanogaster: UAS-20xGCaMP6f*	Bloomington Drosophila Stock Center	BDSC: 42747 RRID: BDSC_42747
*D. melanogaster: UAS-20xGCaMP6f*	Bloomington Drosophila Stock Center	BDSC: 52869 RRID: BDSC_52869
*D. melanogaster: UAS-RSET-jGCaMP8f*	Bloomington Drosophila Stock Center	BDSC: 605083 RRID: BDSC_605083
*D. melanogaster: Gr64f*^*LexA*^	Miyamoto et al.^42^	Flybase: FBti0168176
*D. melanogaster: Gr64f-Gal4*	Dahanukar et al.^43^	Flybase: FBtp0057275
*D. melanogaster: Gr64f-Gal4*	Dahanukar et al.^43^	Flybase: FBti0162678

**Software and algorithms**		

ImageJ	Schneider et al.^44^	https://imagej.nih.gov/ij; RRID:SCR_003
Prism 10	Graphpad	RRID:SCR_002798
Illustrator	Adobe	RRID:SCR_010279

### Experimental model and study participant details

#### Flies

*Drosophila melanogaster* were typically raised on a standard cornmeal diet at 25°C in 70% humidity under a 12-h light/12-h dark cycle unless otherwise stated. For a 30 L preparation of this standard cornmeal diet the recipe is 2127 g D-glucose, 1455 g cornmeal, 909 g torula yeast, 615 g white sugar, 136.5 g agar, 15 g CaCl2-H20, 15 g MgSO4-7H2O and 353 mL of a 50:50 phosphoric/propionic acid mixture. For salt conditioning experiments, flies were placed on food supplemented with 50 mM NaCl for 4 days prior to experimentation. For other diet conditioning experiments flies were kept on indicated diet for 4 days prior to testing. All experimental flies were 4–10 days-old, mated females unless otherwise stated. Genetic manipulations were performed through standard crossing schemes, producing the genotypes listed below for each figure. Further information on source/strain is listed in the key resources table.

#### Fly genotypes by figure

Figure 1:

• *w*^*1118*^
• *+/+; IR56b*^*1*^*/IR56b*^*1*^*; +/+*
• *+/+; LexAop-GCaMP6f/+; Gr64f*^*LexA*^*/+*

Figure 2:

• +/+; *LexAop-GCaMP6f*/+; Gr64f^LexA^/+

Figure 3:

• *+/+; LexAop-GCaMP6f/+; Gr64f*^*LexA*^*/+*
• *+/+; IR56b-Gal4/+; UAS-GCaMP6f/+*
• *+/+; Gr64f-Gal4, UAS-GCaMP6f/+; UAS-IR7c/+*

Figure 4:

• *UAS-GCaMP8f/+; IR56b*^*1*^*/IR56b*^*1*^*; Gr64f-Gal4/UAS-IR56b*
• *UAS-GCaMP8f/+; IR56b*^*1*^*/IR56b*^*1*^*; Gr64f-Gal4/UAS-IR56b*^*TTC*^

Figure S1:

• *+/+; IR56b*^*1*^*/IR56b*^*1*^*; Gr64f*^*LexA*^*/LexAop-GCaMP6m*
• *+/+; IR56b*^*1*^*/+; Gr64f*^*LexA*^*/LexAop-GCaMP6m*

Figure S2:

• *+/+; LexAop-GCaMP6f/+; Gr64f*^*LexA*^*/+*

Figure S3:

• *+/+; IR56b-Gal4/+; UAS-GCaMP6f/+*

Figure S4:

• *+/+; IR56b-Gal4/+; UAS-GCaMP6f/UAS-IR7c*

### Method details

#### Tastants

Tastants used include: NaCl and sucrose, which were kept as 1M stocks and further diluted for pertinent experiments.

#### Calcium imaging

*In vivo* calcium imaging of the GRN axon terminals was carried out as previously described.^17^ Flies aged 4–10 days old were anesthetized briefly with carbon dioxide and placed in a custom chamber. The back of the head was secured using nail polish and wax was applied to either side of the extended proboscis, covering maxillary palps but not touching the labellar sensilla. After 30 min of recovery in a humidity chamber, up to 4 flies had their antennae and a small window of cuticle were removed to expose the SEZ. AHL solution (108 mM NaCl, 5 mM KCl, 4 mM NaHCO_3_, 1 mM NaH_2_PO_4_, 5 mM HEPES, 2 mM CaCl_2_, 8.2 mM MgCl_2_, 15 mM ribose, pH 7.5) was applied immediately. Fly brains were exposed to AHL for up to 20 min prior to imaging. The esophagus was clipped, and air sacs and fat were removed to allow for clear visualization of the SEZ.

GCaMPf fluorescence was captured using a Leica SP5 II Confocal microscope with a 25x water immersion objective. The SEZ was imaged at a zoom of 6-8x, line accumulation of 2, line speed 8000Hz, and resolution of 512 x 512 pixels. The pinhole was opened to 2.86AU. For each taste stimulation, 20–30 total seconds were recorded which included 10 s of baseline, 5 s of stimulation and 5–15 s post-stimulation. A pulled capillary that had been filed down to fit over the labellum, was filled with tastant and positioned close to the labellum using a micromanipulator. For the stimulation, the labellum was immersed in tastant and removed after 5 s. Each fly was stimulated with a particular tastant only once and the stimulator was washed with water between different tastants. Water was used as a negative control tastant and was applied first. Salts were applied in order of increasing concentration and the positive control tastant was applied last.

The maximum change in fluorescence (peak ΔF/F_0_) for peaks was calculated using peak intensity (average of 3 time points) minus the average baseline intensity (10 time points), divided by the baseline. ImageJ was used to quantify fluorescence changes and create heatmaps.

#### Behavioral assays

Binary choice feeding assays were performed as previously described, except flies were not starved prior to the assay to avoid potential confounding effects of starvation.^17^ 4–8 day old flies that had been kept on either regular food or the salt-enriched diet for four days were sorted into groups of 10. Flies were shifted into testing vials that contained six 10 μL drops that alternated between blue (0.125 mg/mL Erioglaucine, FD and C Blue#1) or red (0.5 mg/mL Amaranth, FD and C Red#2) and contained 1% agar along with the 5 mM sucrose. 50 mM NaCl was added to either red or blue dye and approximately half the replicates were done with the dye swapped to control for any dye preference. Flies were given 2 h to feed in the dark at 29°C before being frozen at −20°C. The color of the abdomen was scored as red, blue, purple or no color, using a dissection microscope. Preference Index (PI) was calculated as ((# of flies labeled with tastant 1 color) - (# of flies labeled with tastant 2 color))/(total # of flies with color) and accounted for any flies that were lost in vial transferal and those that did not eat. Any vials with <7 flies or <30% of flies feeding were excluded.

#### Injection protocol

Flies were anesthetized with CO_2_ and given either a sham injection or injected with ∼0.1 μl AHL with no added NaCl (5 mM KCl, 4 mM NaHCO_3_, 1 mM NaH_2_PO_4_, 5 mM HEPES, 2 mM CaCl_2_, 8.2 mM MgCl_2_, 15 mM ribose, pH 7.5) or ∼0.1 μl of AHL with NaCl (100 mM NaCl, 2.5 mM KCl, 2 mM NaHCO3, 0.5 mM NaH_2_PO_4_, 2.5 mM HEPES, 1 mM CaCl_2_, 4.2 mM MgCl_2_, 15 mM ribose pH 7.5) in the abdomen, using the PV830 Pneumatic PicoPump. All flies were allowed to recover for ∼2 h before calcium imaging was performed.

### Quantification and statistical analysis

Statistical analyses were performed with GraphPad Prism 10 software. The figure legend provides the number of biological replicates using different flies for each experiment and the statistical test used. Sample sizes were determined before experimentation based on the variance and effect sizes observed in prior similar experiments. Experimental conditions and controls were run in parallel. Data from calcium imaging experiments were excluded if there was too much movement during the stimulation to reliably quantify the response or if a fly exhibited no response to a known, robust, positive control.
