# Supplementary material for: Modulation of attractive salt taste in Drosophila
Source: iScience. 2026 Feb 24;29(3):115128. doi: 10.1016/j.isci.2026.115128 (PMC12993393; doi:10.1016/j.isci.2026.115128)
Supplement: Document S1. Figures S1–S4 [file mmc1.pdf]

iScience, Volume *29*

Supplemental information

Modulation of attractive salt taste in *Drosophila*

Sasha A.T. McDowell, Jinfang Li, and Michael D. Gordon

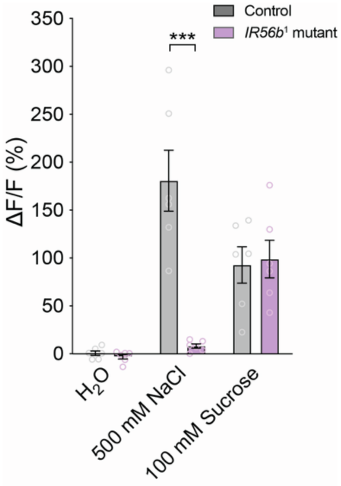

Figure S1: Ir56b is necessary for sodium chloride responses of Gr64f (sweet) neurons. Related to Figure 1.

Calcium imaging of peak Gr64f GRN responses to indicated stimuli. n = 6 flies per group. Bars represent mean +/- SEM. Asterisks indicate significant difference between groups by two-way ANOVA with Sidak's multiple comparison test, ***p < 0.001.

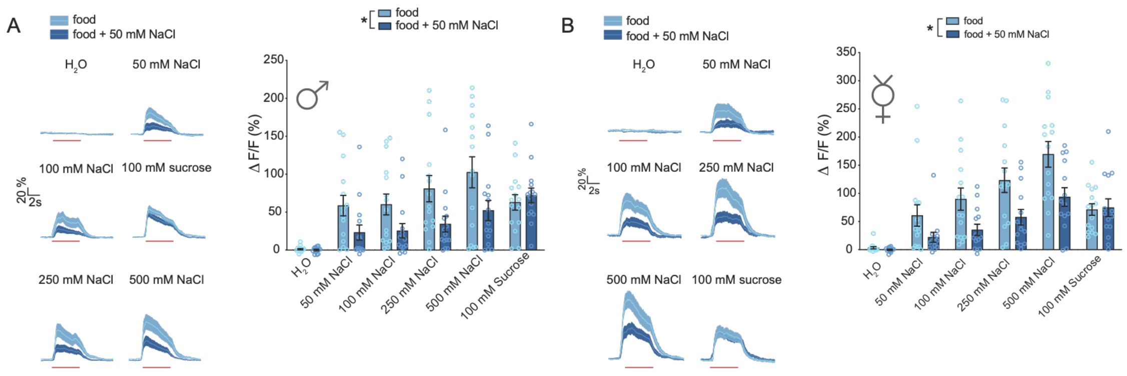

Figure S2: Male and virgin female flies also have modulated Gr64f salt responses. Related to Figure 1.

(A) Calcium imaging of Gr64f GRN responses to increasing NaCl concentrations in male flies kept on food (light blue) and male flies kept on food + 50 mM NaCl (dark blue) prior to imaging, showing time course (left) and peak fluorescence changes (right). n = 15 flies per group. **(B)** Calcium imaging of Gr64f GRN responses to increasing NaCl concentrations in virgin female flies kept on food (light blue) and virgin female flies kept on food + 50 mM NaCl (dark blue) prior to imaging, showing time course (left) and peak fluorescence changes (right). n = 14-15 flies per group. Bars represent mean +/- SEM. Asterisks indicate significant difference between groups by repeated measures ANOVA between food and food + salt groups (B and D); $*p < 0.05$.

A

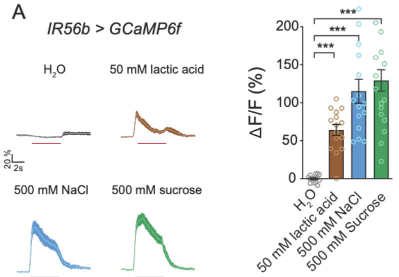

Figure S3: IR56b GRNs respond to 50 mM lactic acid. Related to Figure 2.

(A) Calcium imaging of IR56b GRN responses to 50 mM lactic acid with control tastants H_2O, NaCl and sucrose, showing time course (left) and peak fluorescence changes (right). n = 15 flies per group. Asterisks indicate significant difference between groups by one-way repeated measures ANOVA with Dunnett's post hoc test compared to H_2O. p***<0.0001

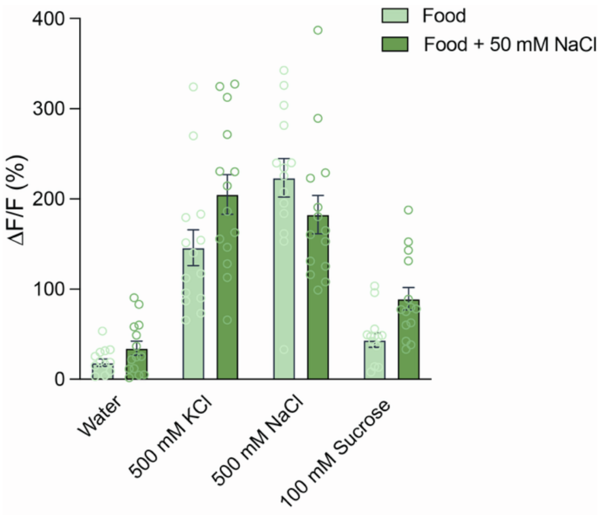

Figure S4: IR7c-dependent potassium current is not modulated in IR56b GRNs. Related to Figure 3.

Calcium imaging of IR56b GRN responses to indicated taste stimuli, showing peak fluorescence changes. Bars represent mean +/- SEM. n = 14 flies per group. No significant differences found by two-way ANOVA with Sidak's post hoc test.
